# Design-build-test of recombinant *Bacillus subtilis* chassis cell by lifespan engineering for robust bioprocesses

**DOI:** 10.1016/j.synbio.2024.04.004

**Published:** 2024-04-11

**Authors:** Kexin Ren, Qiang Wang, Jianghua Chen, Hengwei Zhang, Zhoule Guo, Meijuan Xu, Zhiming Rao, Xian Zhang

**Affiliations:** a Key Laboratory of Industrial Biotechnology of the Ministry of Education, School of Biotechnology, Jiangnan University, Wuxi, Jiangsu, 214122, China; b Yixing Institute of Food and Biotechnology Co., Ltd, Yixing, 214200, China

**Keywords:** *Bacillus subtilis*, Chassis cell, Lifespan engineering, Robustness, Industrial production

## Abstract

Microbial cell factories utilize renewable raw materials for industrial chemical production, providing a promising path for sustainable development. *Bacillus subtilis* is widely used in industry for its food safety properties, but challenges remain in the limitations of microbial fermentation. This study proposes a novel strategy based on lifespan engineering to design robust *B. subtilis* chassis cells to supplement traditional metabolic modification strategies that can alleviate cell autolysis, tolerate toxic substrates, and get a higher mass transfer efficiency. The modified chassis cells could produce high levels of l-glutaminase, and tolerate hydroquinone to produce *α*-arbutin efficiently. In a 5 L bioreactor, the l-glutaminase enzyme activity of the final strain CRE15TG was increased to 2817.4 ± 21.7 U mL^−1^, about 1.98-fold compared with that of the wild type. The *α*-arbutin yield of strain CRE15A was increased to 134.7 g L^−1^, about 1.34-fold compared with that of the WT. To our knowledge, both of the products in this study performed the highest yields reported so far. The chassis modification strategy described in this study can Improve the utilization efficiency of chassis cells, mitigate the possible adverse effects caused by excessive metabolic modification of engineered strains, and provide a new idea for the future design of microbial cell factories.

## High lights

• Lifespan engineering strategy can be used to construct microbial chassis cells.
• The manipulation of cell lifespan can alter cell morphology.
• Systematic modification of cell lifespan is an option for future engineering of *B. subtilis.*

## 1 Introduction

Chassis cell is the core of catalytic production in microbial cell factories [1]. *B. subtilis* is a model strain that is commonly used in industrial fermentation, with a clear genetic background and good workability. It has the ability to use renewable resources to produce various enzymes [2]. In the production application of *B. subtilis*, most of the previous studies focused on the modification of the enzyme and the reconstruction of metabolic pathways. Although these studies have made some progress, the excessive metabolic modifications have led to the slow growth of cells and reduced biomass [3], potentially leading to the premature termination of high-yield periods [4]. Modification of chassis cells simplifies the synthetic biology modification process and demonstrates advantages in the production of a wide range of products. Therefore, it is imperative to establish a desirable *B. subtilis* chassis cell that exhibits optimal robustness, and tolerance for Industrial substrates containing hazardous substances, further leading to the extension of high-yield periods [4]. Previous studies have shown that genome reduction leads to beneficial traits such as genotypic stability and physiological properties stability [5]. In 2016 Li et al. deleted a non-essential region on the chromosome of *B. subtilis* 168, producing 115.2 g L^−1^ of guanosine, which was 4.4 times higher than the WT [6]. Later, Chen et al. found that specific deletion of the *Bacillus licheniformis dlt*ABCD gene, Leads to increased cell membrane permeability, thereby improving mass transfer efficiency, which resulted in a 37.13 % increase in nattokinase activity [7]. In a study by Liu et al., the gene *flgD* knockout strain (A4011) showed a 20 % higher ovalbumin yield than WT strain [8]. These examples demonstrate the superiority of using defective strains for heterologous protein production over standard laboratory models. Therefore, systematic and accurate simplifying the *B*. *subtilis* genome and regulating its growth and production are very meaningful works.

Lifespan engineering provides a platform technology for chassis cell construction, which improves the physiological state of industrial strains and combines metabolic design with the lifespan of microorganisms [9]. The lifespan of *B. subtilis* can be artificially designed although it has evolved over millions of years. The lifespan of microorganisms is divided into the chronological and replicative lifespan [[21], [22], [23]]. Chronological senescence results in decreased cell catalytic efficiency, decreased cell productivity and ultimately lead to cell autolysis. Many studies have shown that autolysis in *B. subtilis* is associated with autolysis enzymes. These autolysis enzymes can be classified into three types based on their relevance: cell growth-associated autolysis enzymes [24], spores-associated autolysis enzymes [25], and prophage-associated autolysis enzymes [26]. Inhibition of cell autolysis is a viable approach to extend the chronological lifespan. There are numerous examples in industrial production where this method has been used to increase production. The anti-autolysis *B. subtilis* LM2531 strain constructed by Wang et al. showed a 1.72-fold increase in whole-cell catalytic β-galactosidase and a 2.6-fold increase in the level of secreted *nattokinase* produced compared with the WT [10]. Zhao et al. combined knockout of peptidoglycan hydrolase-related genes, including *sigD*, *lytE*, *lytF*, *lytC*, *lytD*, and *lytG*, significantly increased cell growth rate and alpha-amylase production [11]. Similar anti-autolysis modification in *Escherichia coli* also yielded satisfactory results [12]. Regulation of the replicative lifespan in *B. subtilis* not only promotes rapid cell growth and alters cellular morphology, but also modulates cell division dynamics. The rate of mass transfer is depending on the specific surface area of the cell [13], while the cell morphology can be partially regulated by the rate of cell division [14]. Cells with smaller surface areas accumulate products more easily, while those with larger surface areas transfer mass more effectively [15]. For example, in the experiments of Jeong et al., they inhibited cell filopodia by overexpressing the gene *ftsZ* and *ftsA* associated with the D phase of cytokinesis, resulting in 57.1 % higher growth density, 30 % higher specific growth rate and 227 % higher production of human leptin protein in *E. coli* [16]. Furthermore, the production rate of industrial fermentation processes can be limited by biomass accumulation. Therefore, selecting fast-growing strains can improve the efficiency of producing industrial chemicals. For example, Hoffart et al. selected the fastest growing non-pathogenic Vibrio *natrivio* as a new chassis to reduce the division time of cells, thereby reducing the division time to 9.4 min and increasing biomass, and alanine production rate to 20 g L^−1^ and 0.56 g L^−1^ ·min^−1^, respectively [17].

**Fig. 1:**
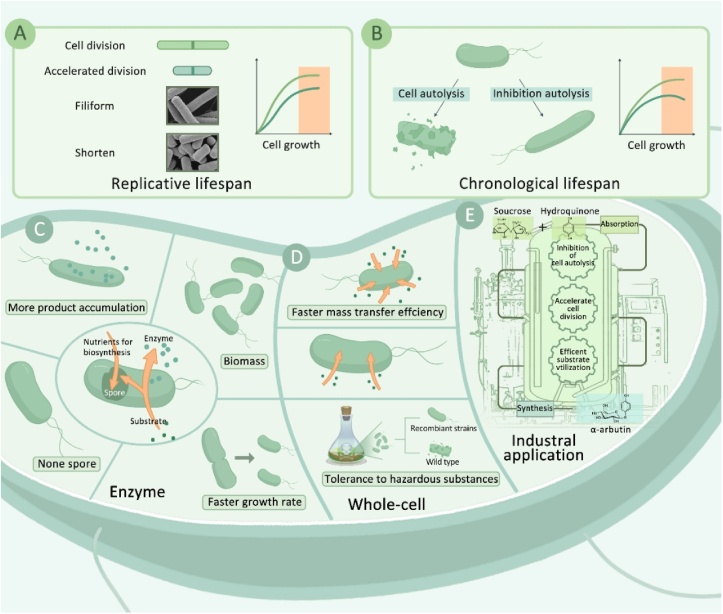
Lifespan engineering-based modification of *B. subtilis* chassis cells for the industrial synthesis of bio-enzymes and whole-cell catalysis. (a) Replicative lifespan-based modification of *B. subtilis* chassis cells to shorten the division cycle and improve biomass and substrate transfer efficiency. (b) Modifying *B. subtilis* based on chronological lifespan to achieve inhibition of cell autolysis at late stages of fermentation by knocking out autolysis-related genes. (c) Modified *B. subtilis* for production of bio-enzymes. (d) Modified *B. subtilis* for production of whole-cell catalysis. (e) Strategies for the industrial production of *α*-arbutin.

In the production of heterologous products such as enzymes, continuous productive activity is a necessity condition for cells, and the process of whole-cell catalysis needs chassis cells' competence for higher mass transfer efficiency and reusability. These requirements can be achieved by modifying the lifespan of chassis cells (Fig. 1). The present study aims to establish a robust *B. subtilis* cell factory with a lifespan engineering strategy. First, the increase in biomass and changes in cell physiological morphology were achieved by regulating the chronological and replicative lifespans of *B. subtilis*, respectively. Then, the energy maintenance coefficients of the different chassis cells and their environmental tolerance were tested to demonstrate that they possessed the basic features in production. Finally, two representative products (l-glutaminase and *α*-arbutin) were expressed using the best strain, achieving the highest yields reported so far. This work is not only a new strategy for the modification of *B. subtilis* but also provides new ideas for the future study of chassis cells.

## 2 Materials and methods

### 2.1 Strains, plasmids, media, and materials

All strains and plasmids used in this study are shown in Table S1. The PCR fragment was assembled by the Clone Expression II One Step Cloning Kit and ligated to the linearised plasmid pMA5-sat. The resulting plasmid was used to transform receptor *E. coli* JM109 cells. All strains were stored at - 80 °C and activated on Luria-Bertani agar plates, and constructed in LB medium at 37 °C. The antibiotics and concentrations used in this study, when required, are as follows: kanamycin, 50 μg mL^−1^, ampicillin, 20 μg mL^−1^ and zeocin, 50 μg mL^−1^ untreated substrate medium: urea 2 g L^−1^, yeast extract 5 g L^−1^, maize pulp 6 g L^−1^, molasses 250 g L^−1^/lignocellulose hydrolysate 150 g L^−1^.

### 2.2 Construction of chassis strains

The knockout method used was described in a previous study [18]. All fragments, including the upstream sequence (800 bp), the *lox*71-*zeo*-*lox*66 fragment, and the downstream sequence (800 bp), were ligated using fusion PCR. DNA polymerases used for PCR is 2 × Phanta Max master mix (Vazyme Biotechnology CO., Ltd.). The purified PCR product was then used to transform receptor *B. subtilis*, followed by a selection of transformants resistant to Zeo^r^. The Cre/*lox* system was used to remove the resistance marker from the host strain.

### 2.3 Physiological analysis

The Origin software was used to fit the growth curve, calculate the specific growth rate (μ), and take the maximum value to represent the specific growth rate of the strain. S-functions were selected for nonlinear curve fitting. The growth rate of the original bacteria was calculated to be 0.51**·**h^−1^. PI staining: The cells were collected at approximately 10^6^ cells**·**mL^−1^ and the supernatant medium was discarded. The cells were then washed once with 3 mL of PBS and resuspended into PBS. 1 mL PI was used to stain the cells for 20 min at 4 °C, protected from light. Flow cytometry experiments: Argon ion excited fluorescence was used with a laser wavelength of 488 nm and an emission wavelength of >630 nm to produce a red fluorescence to analyze the histogram of the PI fluorescence intensity and also to analyze the scatter plot of the scattered front light against the side scattered light. Unstained cells were used as a negative control for cell autofluorescence. For each sample, a minimum of 20,000 counts were recorded using a flow rate of 0.5 mL s^−1^. Determination of viable cell counts: The *B. subtilis* solution cultured to a stable stage was diluted to 10^5^, samples were applied to the plates, and each sample was replicated 3 times and the number of viable colonies on the plates was counted as the number of viable bacteria in the sample.

### 2.4 Strain maintenance energy coefficient and dry weight calculation

Each strain was incubated continuously in a fermenter containing 0.4 L (LioFlo110, New Brunswick Scientific, USA). The fermenters were inoculated by incubating in 50 mL seed medium. When the glucose was almost depleted, the culture was switched from batch to chemostat mode, maintaining the pH at 6.6. The aeration rate, agitation rate, and temperature were controlled at 450 rpm, and 37 °C. The dilution rate (D) was gradually increased from 0.1 h^−1^ to 0.5 h^−1^. The growth of cells eventually reached a steady state, with both cell density and glucose consumption rates remaining constant over time. Cell growth was monitored using a spectrophotometer, while samples were collected and dried to a constant weight at 80 °C before being weighed. Meanwhile, glucose consumption was continuously monitored throughout the experiment. The experimental value for the maintenance energy coefficient was expressed as the rate of glucose consumption (m_glc_).

### 2.5 l-glutaminase fermentation process and enzyme activity assay of recombinant strains

For the shake flask phase, the recombinant strains were activated and inoculated into a 50 mL flask containing 20 μg**·**mL^−1^ Kan^r^ at 3 % inoculum volume. The plates were incubated at 30 °C for 30 h, centrifuged and washed with PBS (pH 7.5). The bacterial suspension was then sonicated for 20 min after mixing with 5mLPBS containing 50 μL lysozyme (1g**·**L^−1^). Finally, the mixture was centrifuged at 10,000 rpm for 30 min to separate cell debris. After centrifugation, the resulting supernatant was a crude l-glutaminase solution, which was used to measure l-glutaminase activity. For testing in the middle stage of a 5 L bioreaction, recombinant strains were inoculated with 100 mL of seed culture into a 5 L bioreactor containing 2 L of fermentation medium after a two-stage seed expansion culture. The DO-stat fed-batch fermentation strategy was used for fermentation.

The l-glutaminase activity was determined by measuring the amount of l-glutamate produced, following a 5-min incubation at 55 °C and subsequent termination with 80 μL of 20 % trichloroacetic acid. The reaction mixture (1 mL) contained 900 μL of 200 mM l-glutamine and 20 μL of l-glutaminase. The concentration of l-glutamate in the supernatant obtained after centrifugation was determined by a biosensor analyzer (Jinan Yanke Instrument Co., LTD.). One unit (U) of l-glutaminase activity is the amount of enzyme required to produce 1 μmol l-glutamic acid per minute.

### 2.6 Method for whole-cell catalytic production of *α*-arbutin

The whole cell catalytic process is anaerobic and dark to minimize the toxic substances such as benzoquinone produced by hydroquinone oxidation. The transformation system was 10 mL, pH 7.0, and reaction temperature 30 °C. Refer to Ao et al. [19] for details of the procedure. The 5 L bioreactor was used for cultivation at 10 % inoculum. The stirring speed increased gradually from 220 to 600 rpm, and after 24 h, the cells were collected for whole-cell biotransformation. Cells, 40 g L^−1^ hydroquinone and 400 g L^−1^ sucrose were simultaneously added to 50 mM PB buffer (pH 7.0), and 10 g L^−1^ and 6 g L^−1^ hydroquinone were added at the 3rd and 8th hour, respectively.

### 2.7 Determination of *α*-arbutin production

The concentration of *α*-arbutin was determined on an Agilent LC 1260 HPLC system using Dima Technologies Diamonsil C18(2) column (5 μm 250 × 4.6 mm), UV detector set at 281 nm, the mobile phase consisted of 10 mmol L^−1^ dilute phosphoric acid and methanol, the volume ratio of 4:1, the flow rate of 0.5 mL min^−1^. The column temperature was controlled at 35 °C and the injection volume was 10 μL.

### 2.8 Cell counting and specific surface area calculation

The cell model of *B. subtilis* 168 was used to calculate the mean cell diameter and mean cell length. Morphological parameters of *B. subtilis* 168 were identified by cell contouring of 100 individual cells in Nikon 80i microscope images. The specific surface area is calculated by dividing S (1) by V (2). S = 2πR (L-2R) + 4πr^2^ (1), V = πr^2^ (L-2R) + 4/3 πr^3^ (2). In the formula, R is the average cell diameter, L is the average cell length, V is the cell volume, and S is the surface area [20].

For fluorescence microscopy, 2.5 μL of culture sample is placed on a microscope slide coated with a thin agarose (1 %) layer covering the slide. For membrane or DNA staining, 10 μL of the sample was mixed with 1.0 μL of Nile Red (molecular probe), 12 μg mL^−1^ or 1.0 μL of DAPI solution in 50 % glycerol, respectively. Cell length measurements were performed using METAMORPH 4.6.9 software (Universal Imaging).

### 2.9 Statistical analysis

The data in this study represent the mean and standard deviation of multiple independent experiments. Significant differences in the data were determined by one-way ANOVA. 0.01 < P < 0.05 difference significant, P value < 0.01 difference significant.

## 3 Results

### 3.1 Chronological lifespan engineering of recombinant ***B. subtilis*** chassis cells

**Fig. 2:**
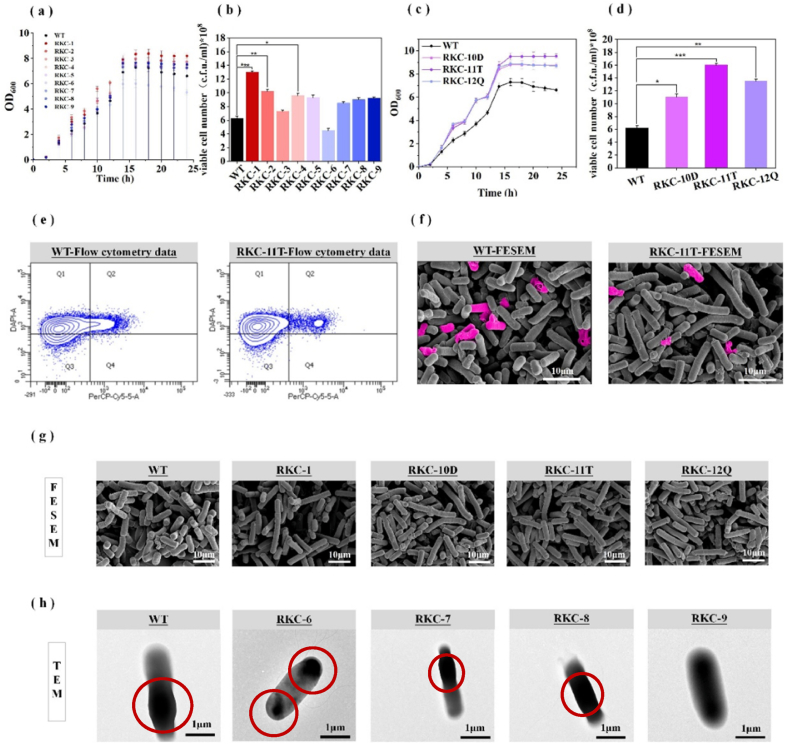
The result and analysis of chassis cell engineered by chronological lifespan. (a) Growth curve of single knockout strain (RKC-1-RKC-9). (b) Diluted coated plates of single knockout strain counting live cells (24h, LB medium). (c) Growth curve of multi knockout strain (RKC-10D, RKC-11T, RKC-12Q). (d) Diluted coated plates of multi knockout strain counting live cells. (e) Survival of WT versus recombinant RKC-11T cells at late stages of flat growth, the number of dead cells detected by PI-stained flow cytometry. (f) WT and strain RKC-11T FESEM was photographed and images were analyzed using Adobe Photoshop 6.0. In the picture, dead cells that have been damaged by autolysis are marked in pink. (g) Morphological changes of the recombinant strains. (h) Distribution and changes of spores, plotted from left to right, WT, RKC-6- 9.Spores are highlighted using red circles.

Firstly, we constructed a series of chassis strains resistant to autolysis and tested for their growth (Fig. 2a). Knocking out the growth-related autolysis genes *lytC, sigD, pcfA* [27] and *flgD* in *B. subtilis* 168 (WT) produced strains RKC-1,RKC-2,RKC-3, and RKC-4, which biomass (OD_600_) increased by 20, 17, 12, and 11 %, respectively. The prophage-associated gene *xpf* knock-out strain RKC-5 increased by 10 % in biomass, and strains RKC-6-9 with knockout of the spore-associated global regulatory transcription factors *spo0A* and spores-associated autolysis enzymes genes *skfA, sdpC* and *spoIIE* produced 20 % reduction and 10, 8, 14 % increases.

For the genes *lytC*, *sigD*, *pcfA*, and *flgD* that were screened according to the above experiments and had great influence on cell biomass, we carried out one by one combination knockout and obtained the strains RKC-10D, RKC-11T, RKC-12Q. Although the maximum biomass of *spoIIE* is increased by 14 %, its autolysis effect is still obvious. Thus this gene was not selected for further study. The biomass of strains RKC-10D and RKC-11T increased 32 % and 38 %, respectively, while the biomass of strain RKC-12Q increased by only 30 % (Fig. 2c). The results showed that the resistance of the strain to autolysis did not increase with the number of related autolysin genes knocked out. Excessive knockout of the functional genes may cause unpredictable or cascading impairments in cell function. As OD_600_ only reflects cell quantity and not viability, this study examined the number of viable cells to confirm whether the aforementioned manipulations extended cell lifespan. The results depicted in Fig. 2d reveal that strain RKC-1 and RKC-11T exhibited a 2.1-fold and 2.8-fold increase in number of viable cells, respectively, compared to the strain WT. To verify the survival of cells in the later stages of fermentation, shake flask cultures of cells in the stable growth phase were examined by electron microscopy. The relative survival rates of WT and RKC-11T reached 68.6 % and 88.0 %, respectively (Fig. 2e and f). Combining all the above results, strain RKC-11T can be used in subsequent research.

Interestingly, the impact of certain genes on the morphology of *B. subtilis* was observed through scanning electron microscopy, transmission electron microscopy (TEM), and field emission scanning electron microscopy. Compared to the WT strain, the deletion of gene *lytC* resulted in a cell length that was approximately 4.5 times longer than that of the WT strain (Fig. 2g). Additionally, the deletion of genes *spo0A* and *spoIIE* led to cell-cell polarization and loss of spores, while no changes were observed with deletion of genes *skfA* and *sdpC* (Fig. 2h).

### 3.2 Replicative lifespan engineering of recombinant ***B. subtilis*** chassis cells

Manipulating the replicative lifespan of cells can accelerate cell division, thereby partially delaying cell senescence and ultimately increasing biomass production [28]. The bacterial division and cell wall (dcw) cluster is a highly conserved region of the genome that encodes several essential cytokines, including the cell division protein FtsZ [29] and FtsL [30], which are associated with Z-loop maturation and facilitates the subsequent division process. In *B. subtilis*, overexpression of gene *mraZ* or lack of FtsL results in the decondensation of the FtsZ loop (Z-loop) [31], preventing cells from dividing. Therefore, the zinc metalloprotease YluC, which is required for the turnover of genes *mraZ* and *ftsL* [32,33], was selected as the main subject of this study. It has been shown that mutants lacking gene *yluC* in *E. coli* have altered morphology and tend to be approximately 33 % shorter than the WT, further suggesting that gene *yluC* deletion promotes faster cell division [32].

**Fig. 3:**
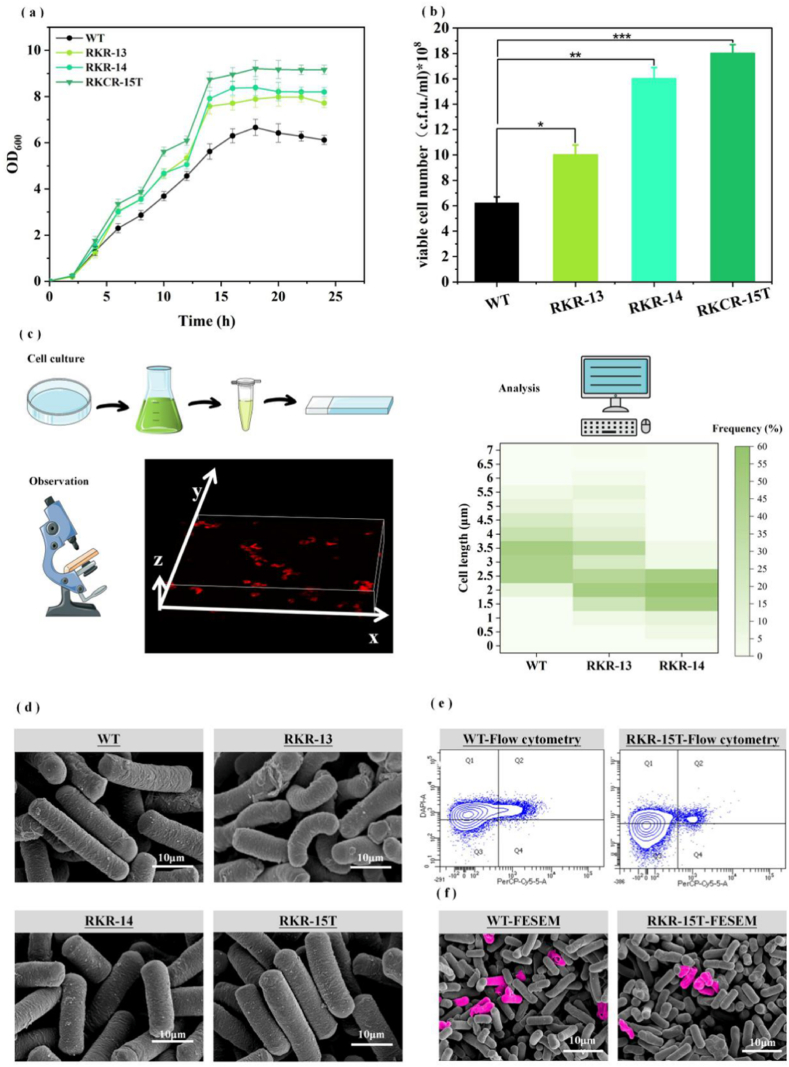
The result and analysis of chassis cell engineered by replication lifespan. (a) Growth curve of recombinant strain (24 h, LB medium). (b) Dilution of the recombinant strain on coated plates to count live cells. (c) Cell length distribution in mutants. The cells show length distribution in CH medium (1 mM IPTG), membranes stained with Nile Red prior to length measurement. (d) Morphological changes of recombinant strains. (e) Survival of WT versus recombinant RKR-15T cells at late stages of flat growth, the number of apoptotic cells detected by PI-stained flow cytometry. (f) WT and strain RKR-15T FESEM was photographed and images were analyzed using Adobe Photoshop 6.0. In the picture, dead cells are marked in pink.

To extend the replicative lifespan of chassis cells, this study first generated the *yluC* knockout strain RKR-13, and the *mraZ* knockout strain RKR-14, and determined their growth curves and specific growth rates. The specific growth rate of strains RKR-13, RKR-14 and RKCR-15T reached 0.78·h^−1^, 0.85·h^−1^, 0.92·h^−1^, increased by 56.11 %, 70.04 % and 84.8 % compared with WT (μ = 0.51·h^−1^), respectively. This indicates that the recombinant strain is dividing at an accelerated rate (Fig. 3a). Knocking out gene *yluC* in *B. subtilis* 168 (RKR-13) showed about a 10 % increase in the number of viable cells (Fig. 3b), and a shortening of the length of the cells as observed by microscopy. The knockout of gene *mraZ* in WT (RKR-14) resulted in a reduction in cell length (Fig. 3c) and an increase of 23 % in OD_600_ compared to the strain WT (Fig. 3a). During the subsequent work of constructing the double knockout strain of *mraZ* and *yluC*, it was found that there were no positive transformants on the Petri dish, so we hypothesized that the co-knockout of these two genes might cause strain death. Therefore, *mraZ* gene was selected for further research and knocked it out in the RKC-11T resulting in strain RKCR-15T which exhibited superior anti-autolysis ability with a maximum value of 97.0 % (Fig. 3e and f). These results indicate that lifespan engineering manipulations are effective in increasing cell growth rate and cell survival. After observing the morphology of the modified strain, we found that without gene *mraZ*, the cell length was significantly reduced, the diameter was increased and the lack of *yluC* gene resulted in a distorted and folded cell shape. The graph illustrates that the specific surface area (SSA) of strain RKR-13 increased to 13.2 μm^−1^, and RKCR-15T increased to 12.9 μm^−1^ (11.5 μm^−1^ for the WT), resulting in a significant enhancement of substrate transfer efficiency. In addition, our observations with strain RKCR-15T revealed that its diameter was increased (Fig. 3d), which was the key reason for its increased specific surface area.

### 3.3 Analysis of the maintenance energy coefficient of ***B. subtilis* chassis cells**

The maintenance energy coefficient is a physiological parameter that reflects the energy required to maintain intracellular homeostasis [34], a low maintenance energy metabolism testifies to a high energy utilization efficiency in chassis cells. *B. subtilis* has a higher maintenance energy coefficient, which is a significant reason that prevents it from being the optimal chassis cell [35]. Therefore, it is vital to reduce unnecessary metabolic consumption during cell growth. The four strains selected from the pre-experiment RKC-1, RKC-11T, RKR-14, RKCR-15T, with strong growth ability, were tested for the maintenance energy coefficient. All of the knockout strains showed a trend toward a lower coefficient than the control strain. The control strain (*B. subtilis*168) had the highest coefficient of 0.4 mmol g (cdw)^−1^ h^−1^, which was reported to be 0.39 [36]. To further reduce the coefficient, this study knocked out gene *sigE* (RK-E), which regulates the third stage of sporulation [36], in WT and all the above strains (RKE, RKCE1, RKCE11, RKRE14, RKCRE15). Because the absence of non-essential genes causes cells to spend less energy on cell processes unrelated to growth, there is a tendency for the maintenance energy coefficient of the recombinant strains to decrease [37]. Strain RKCE11 had the lowest coefficient, 0.33 mmol g (cdw)^−1^ h^−1^, this was a 15 % decrease compared to the control strain (Table 1).

**Table 1:** Comparison of growth-related data of genome-reduced strains RKE, RKCE1, RKCE11, RKRE14, RKCRE15 and the WT under aerobic conditions.

Strains	WT	RKE	RKCE1	RKCE11	RKRE14	RKCRE15
Maintenance coefficient mmol·g (cdw)^−1^ h^−1^	0.40 ± 0.03	0.35 ± 0.02	0.35 ± 0.03	0.33 ± 0.03	0.36 ± 0.01	0.34 ± 0.02
CDW (g·L^−1^)	1.59 ± 0.01	1.76 ± 0.03	1.98 ± 0.02	2.16 ± 0.02	2.06 ± 0.03	2.43 ± 0.01
Glucose consumption (g·L^−1^)	6.55 ± 0.11	6.15 ± 0.15	6.22 ± 0.12	6.05 ± 0.12	6.35 ± 0.10	6.74 ± 0.11
Biomass yield (g·g^−1^glucose)	0.25 ± 0.03	0.28 ± 0.02	0.30 ± 0.04	0.33 ± 0.04	0.34 ± 0.01	0.35 ± 0.01

In addition, the dry cell weight of RK series strains also changed, with strains RKCE11 and RKCRE15 exhibiting increases in dry weight by 35.8 % and 52.4 %, respectively. Due to their lower glucose uptake and higher biomass, the recombinant strains also demonstrated an increase in biomass yield.

### 3.4 Tolerance test of *B. subtilis* chassis cells to toxic substances

**Fig. 4:**
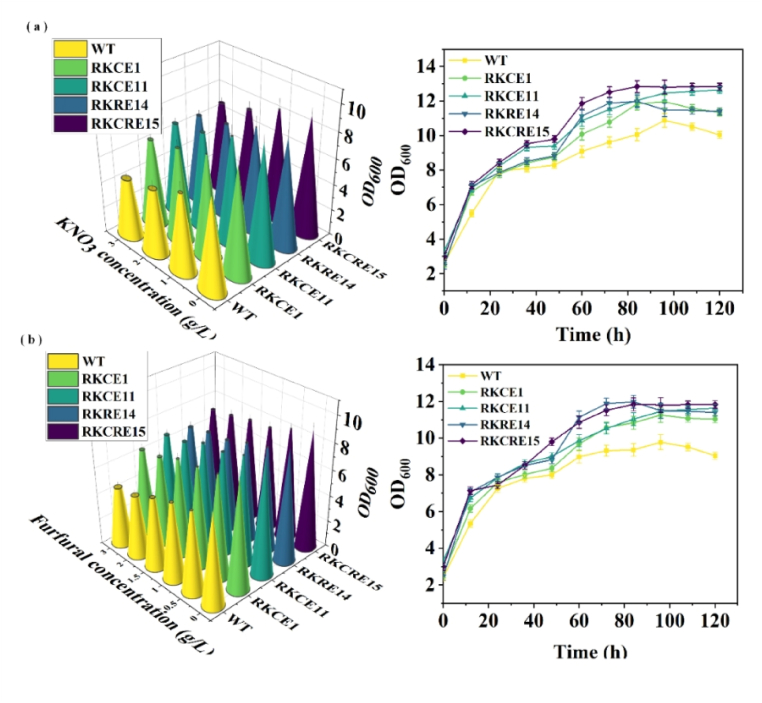
Growth curve of the strain in the untreated substrate fermentation medium. (a) Pre-experiment on the effect of nitrate on the growth of *B. subtilis* and determination of the growth characteristics of recombinant chassis cells in industrial molasses untreated culture medium. (b) Pre-experiment on the effect of furfural on the growth of *B. subtilis* and determination of the growth characteristics of recombinant chassis cells in lignocellulose hydrolysate medium.

This study further researched whether the modified chassis cells could show greater adaptability than the control in extreme environments, which is an excellent trait that is required for industrial production. Efforts have been made to continuously increase yields and explore the feasibility of utilizing raw materials that are either free or low-cost [38,39]. The untreated substrates are essentially unprocessed and contain essential nutrients for microbial growth, but also impurities that can hinder microbial growth, such as aldehydes and alcohols. Common untreated substrates include maize pulp, molasses, bagasse, lignocellulose hydrolysates, and even banana skin [40], pig manure, etc. [41]. The most prevalent harmful impurities include the non-protein nitrogen source, nitrate, and aldehydes. Typically, untreated substrates such as spent molasses and lignocellulosic hydrolysates have harmful matter content in the range of 0.5–2.0 w/w. Pre-experiments involving recombinant strains were conducted to test for tolerance. Firstly, media with potassium nitrate concentrations of 0 g L^−1^, 1.0 g L^−1^, 2.0 g L^−1^, and 3.0 g L^−1^ and furfural concentrations of 0 g L^−1^, 0.5 g L^−1^, 1.0 g L^−1^, 1.5 g L^−1^, 2.0 g L^−1^, and 3.0 g L^−1^ were selected to test the tolerance of hazardous substances. The results showed that the cell biomass (smooth growth OD_600_) decreased by 10–20 % when WT was incubated between 0.5 g·L^−1^and 2.0 g·L^−1^harmful matter content. While at 3.0 g L^−1^, this value reached 35.4 % (in media with potassium nitrate) and 38.5 % (media with furfural) (Fig. 4a), As the concentration of harmful substances increased, the cell vitality gradually decreased (Table S2). This suggests that the presence of nitrate-like impurities in the untreated substrate has a severe impact on cell growth. The results of recording the growth of strains grown in shake flasks for 12–120 h in untreated substrate fermentation medium are shown in Fig. 4b. It was found that the OD_600_ of recombinant strains all increased, strain RKCRE15 had the highest increase, 20 %. This result demonstrated that lifespan engineering modifications not only prolonged cell lifespan and fermentation cycles, but also enhanced the tolerance of chassis cells to untreated substrates.

### 3.5 Comparative tests of l-glutaminase production of recombinant strains

**Fig. 5:**
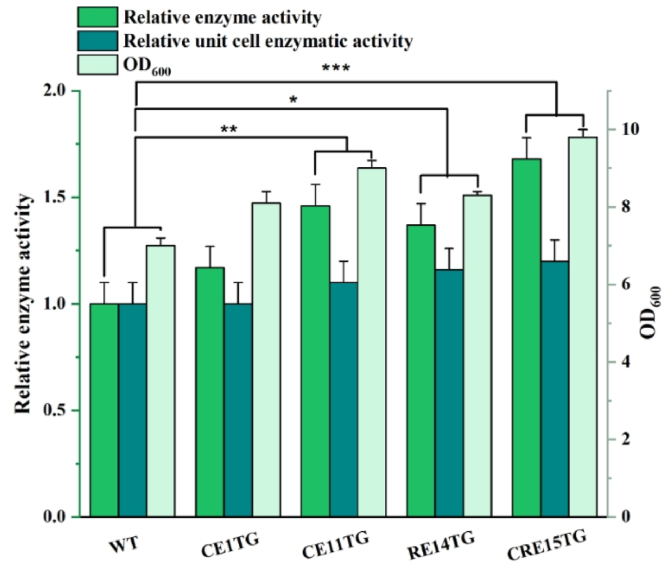
Detection of enzyme production ability of recombinant chassis cells. Glutamine aminotransferase relative enzyme activity, relative cytosolic enzyme activity, and OD_600_. (The recombination strains CE1TG, CE11TG, RE14TG, CRE15TG, which were constructed from RKCE1, RKCE11, RKRE14, RKCRE15,with pMA5-*glsA*).

To assess the enzyme production capacity of the engineered chassis cells, this study evaluated an enzyme commonly produced by *B*. *subtilis*, l-glutaminase, using the recombination strains CE1TG, CE11TG, RE14TG, CRE15TG, which were constructed from the four chassis strains that performed best in the above tests. The strains CE1TG and CE11TG exhibited a significant advantage in product accumulation, with an increase of 18 % and 30 % in OD_600_ at the end of fermentation compared to the WT. Additionally, these cells demonstrated approximately a 4.5 times increase in length, indicating their remarkable ability to accumulate enzyme products. The l-glutaminase enzyme activities in strains CE1TG and CE11TG increased from 16.32 ± 0.56 in the WT to 20.56 ± 2.01 and 22.43 ± 1.75 U mL^−1^, and an increase of 10 % and 16 % per unit OD_600_, respectively (Fig. 5). The strains RE14TG and CRE15TG exhibited enhanced biomass and enzyme production, with the OD_600_ of RE14TG and CRE15TG reaching 1.25 and 1.37 times that of the WT at the end of fermentation, while enzyme activity increased by 37.6 % and 68.7 %, respectively. This finding further confirms a linear correlation between the enzyme production and biomass of the recombinant strains, with specific growth rate and mass transfer efficiency being identified as potential secondary limiting factors.

### 3.6 l-glutaminase production by the recombinant strain CRE15TG during fed-batch fermentation

**Fig. 6:**
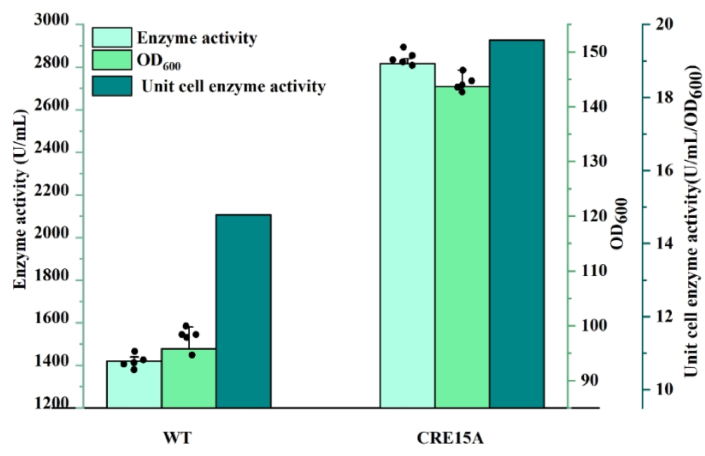
Data related to fermentation products l-glutaminase in the 5 L bioreactor.

l-glutaminase is a growth-related product in bioreactor cultures, therefore, the final strain CRE15TG was more suitable for its production than the strain WT. Stirred tank reactors with automated control systems were selected to use in this study. During fermentation, the speed and DO coupling were controlled at 30 %, and the upper limit of the speed was set to 1000 rpm (Fig. S1). Finally, the l-glutaminase activity of CRE15TG (2817.4 ± 21.7 U mL^−1^) was increased by 98.4 % compared with that of the WT strain (1422.9 ± 19.56 U mL^−1^). The OD_600_ of the strain WT and the recombinant strain were 95.8 and 143.7, respectively, and the average yield per unit OD_600_ was 14.8 U mL^−1^ and 19.6 U mL^−1^, respectively (Fig. 6). As mentioned above, CRE15TG exhibited a lower maintenance energy coefficient and more biomass within a specific bioprocess, demonstrating that the high biomass was the key factor for the high l-glutaminase production of this recombinant strain. It is worth mentioning that, the yield of this work was 11 % higher than the reported highest yield of 2516.8 U mL^−1^ obtained by optimization of the 5′-UTR sequence in our previous study [42].

### 3.7 Whole-cell catalytic efficiency testing of recombinant strain

**Fig. 7:**
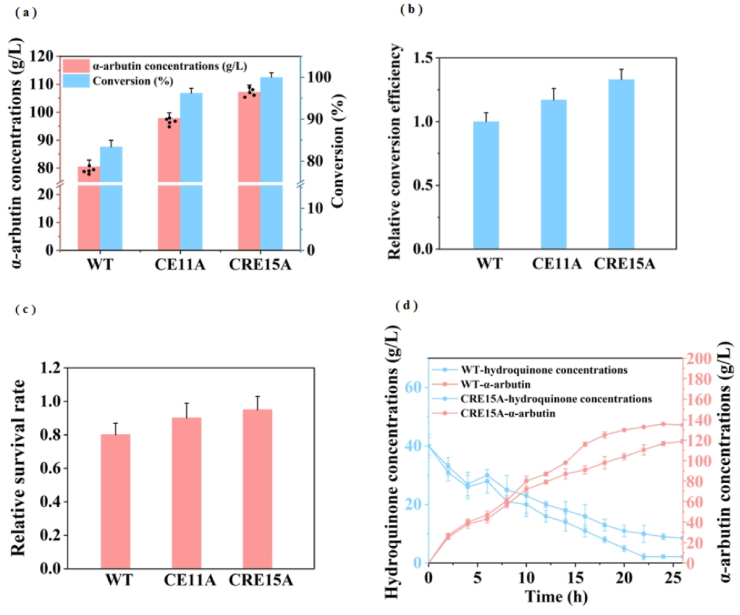
Detection of whole cell catalytic ability of recombinant chassis cells. (a) Yield and conversion concentration of whole-cell catalytic *α*-arbutin production by recombinant strains, repeat the parallel experiment five times. (The recombination strains CE11A, CRE15A, which were constructed from RKCE11, RKCRE15,with pMA5-*gtfA*_R137F_). (b) The relative conversion efficiency of *α*-arbutin. (c) Cell lysis after the use of 3 times whole-cell catalysis. (d) Production of *α*-arbutin and the consumption of hydroquinone in the 5 L fermenter during 24 h fed-batch biotransformation.

In complex microbial industrial fermentation environments, many of the substrates used through whole-cell catalysis are toxic to the cell and the enzyme. For instance, hydroquinone can cause cell lysis while exposing intracellular enzymes to a toxic environment resulting in decreased enzyme activity and ultimately reduced *α*-arbutin production. Previous experiments have found that the knockout of the autolysis-related gene *lytC* can improve the production of *α*-arbutin [19]. Therefore, in this study, we further studied the tolerance of chassis cells to toxic substances and improved the mass transfer efficiency of substrates. Based on the above detection of the enzyme production ability of the recombinant strain, this study constructed strains CRE11A and CRE15A from strains RKCRE11 and RKCRE15 which carried plasmids containing the modified sucrose phosphorylase gene. The recombinant strains were transformed under the same conditions as the strain WT by shaking the flask culture (Fig. 7a), strain CRE15A produced 1.37 times (107.2 g L^−1^) *α*-arbutin higher than the strain WT (80.5 g L^−1^). In addition, the conversion efficiency of substrate hydroquinone has been significantly enhanced. In the preliminary experiments, the cessation time of strain WT transformation was 24 h, and the concentration of transformation substrate was 40 g L^−1^. In the process of catalysis, samples were taken every 4 h, and it was found that the hydroquinone of the recombinant strain was almost exhausted within 20 h and CRE15A completed the catalysis at 16 h, and the conversion efficiency was increased by 33 % (Fig. 7b). The autolysis rate detected in the cell after whole-cell catalysis is shown in Fig. 7c. The OD_600_ of the WT strain decreased by approximately 20 %, whereas the recombinant strain exhibited a significantly lower decrease rate than that of the WT, with CRE15A showing only a 5 % reduction in OD_600_. This provides a great advantage for the reuse of whole-cell catalysis at later stages.

### 3.8 Batch feeding anaerobic whole-cell biotransformation of *α*-arbutin in a bioreactor

The biomass of cells cultured in a shaking flask was too low to achieve the optimal amount of whole-cell catalytic reaction, so a 5 L bioreactor was used to culture the cells. To further increase the yield of *α*-arbutin, this study changed the substrate addition to batch feeding during whole-cell catalysis. After adding cells and substrates under optimal reaction conditions, the reactor was sealed to ensure the anaerobic environment required for the reaction (Fig. 7d). After transformation, it was found that the yield of *α*-arbutin of the final recombinant strain CRE15A reached 134.7 ± 1.5 g L^−1^, which was 14 % higher than that of the WT strain. It has been previously reported by our group that the highest yield of *α*-arbutin was 129.6 g L^−1^ [43]. This study replaced the chassis cells based on previous work and achieved not only higher yield but also higher conversion efficiency per hour by 13 % (Table 2). The optimized batch feeding method alleviated the negative effects of substrate on cells, and the recombinant strain CRE15A showed higher *α*-arbutin yield under the same OD_600_, which further indicated its tolerance to toxic substrates.

**Table 2:** Relevant data at the end of batch anaerobic whole cell bioconversion in a bioreactor.

Strains	*α*-arbutin (g·L^−1^)	Hydroquinone (g·L^−1^)	Reaction completion time (h)	Conversion efficiency (g·L^−1^·h^−1^)	Rate of cell loss (%)	Molar conversion rate (mol·mol^−1^)
WT	118.7 ± 1.3	8.5 ± 1.5	26	1.91 ± 0.10	14	0.86 ± 0.01
CRE15A	134.7 ± 1.5	2.2 ± 0.7	22	6.09 ± 0.10	5	0.97 ± 0.01
Previous study(Ao et al., 2023a)	129.6 ± 2.0	6.9 ± 1.1	24	5.31 ± 0.11	11	0.95 ± 0.01

## 4 Discussion and conclusion

The development of efficient green bio-manufacturing technology based on excellent microbial chassis cell performance is a circular economy transformation technology with great application potential [44]. Our objective in this study was engineered microbial chassis cells from the physiological perspective, and compared with previous studies, the engineering strategy in this study systematically analyzed the effects of chassis cell lifespan and morphology on industrial production. A lifespan engineering strategy was designed to engineer *B. subtilis* chassis cells in this study, which enabled a significant yield improvement in bio-manufacturing without the need for overexpression element modification and metabolic engineering. Firstly, we selected and knocked out nine key autolysin-related genes to resist autolysis. In this step, we found that it was difficult to achieve increased linearly strain biomass whith the number of relevant knockout genes, therefore, it was most convenient to continue the assay with the double knockout strain. We further knocked down the *Z*-loop regulator *mraZ* [31] to alleviate cell division inhibition. This systematic manipulation of the lifespan of *B. subtilis* resulted in the prolongation of both the replicative and chronological lifespans of the cells. In the process, the morphological changes of the cells were also observed. Knockout of the *lytC* gene resulted in a 4 to 5-fold increase in cell length, a change that resulted in more efficient accumulation of intracellular products. Knockout of *mraZ* reduced the length of the cells to 60–70 % of the original cells and increased the diameter by 20–30 %. The increase of the SSA of the cells further enhanced the mass transfer efficiency, which was the key to the improved whole-cell catalytic efficiency. More interestingly, we found that the combined knockout of these two genes left the cell length unchanged and the diameter increased, so we speculated that the factors affecting cell morphology were additive.

The extended of lifespan prevents the development of cell aging of industrial strains and reflects higher cellular toxicity resistance and robustness in untreated and toxic substrates, protecting intracellular enzymes from inactivation by toxic substrates such as hydroquinone. In the presence of furfural and nitrate (0.5–2.0 %), two common untreated substrate toxic substances, strain RKCRE15 still maintained a high growth rate and biomass, while the WT was severely inhibited. This advantage makes the culture cost of *B. subtilis* chassis cells much lower.

Production-oriented microbial chassis cells need to have a low energy maintenance factor in order to apply more energy to production. Knockout of gene *sigE*, a sporulation stage three regulator [45], precisely reduced the energy consumption for sporulation, with no loss of growth and survival compared to the previous sporulation-free chassis derived by *spo0A* deletion [46].

This new chassis cell not only solved the problem of autolysis at the later stage of the fermentation process but also further increased the biomass and prolonged the period of high cell yield, making it more suitable for high-density long-term fermentation (Fig. S1). The modified chassis cells could produce high levels of l-glutaminase and tolerate hydroquinone to efficiently produce *α*-arbutin.

Although we have produced two typical microbial products using chassis cells and achieved promising results, more products of different types are still needed to test their versatility. In the fermentation of the growth-associated product l-glutaminase, the non-growth-associated product was found to have a more significant increase in yield at the later stage of fermentation (unpublished data). On the other hand, when regulating the replication lifespan of *B. subtilis*, we found that the actin-like protein gene *mreB* [47] also has a certain regulatory effect on it, and the regulation of its expression level and expression time may be the key to the improvement of chassis cells. Improvements are currently under study to complete the system, including testing for the production of non-growth-related products, testing for whole-cell catalysis with high substrate concentrations, semi-rational design to regulate *mreB* expression and enhance cell temperature and pH resistance, etc. These strategies will further improve the industrial performance of *B. subtilis* chassis cells and achieve large-scale production to meet market demand. This study not only provides a reference for the engineering route of *B. subtilis*, but also provides a feasible idea for the construction of microbial cell factories from a new perspective.

## Notes

The authors declare no competing financial interest.

## Ethical statements

This article does not contain any studies with human participants or animals performed by any of the authors.

## Data availability statement

All data that support the findings of this study are included within this paper and its Supplementary Information files.

## CRediT authorship contribution statement

**Kexin Ren:** Investigation, Visualization, Writing – original draft, Writing – review & editing. **Qiang Wang:** Writing, Visualization. **Jianghua Chen:** Investigation. **Hengwei Zhang:** Investigation. **Zhoule Guo:** Investigation. **Meijuan Xu:** Writing – review & editing. **Zhiming Rao:** Supervision. **Xian Zhang:** Conceptualization, Supervision, Funding acquisition.

## Declaration of competing interest

The authors declare that they have no known competing financial interests or personal relationships that could have appeared to influence the work reported in this paper.
