# Supplementary material for: Design-build-test of recombinant Bacillus subtilis chassis cell by lifespan engineering for robust bioprocesses
Source: Synth Syst Biotechnol. 2024 Apr 11;9(3):470–80. doi: 10.1016/j.synbio.2024.04.004 (PMC11021899; doi:10.1016/j.synbio.2024.04.004)
Supplement: Multimedia component 1 [file mmc1.docx]

**Supporting Information**

Design-build-test of recombinant *Bacillus subtilis* chassis cell by lifespan engineering for robust bioprocesses

Kexin Ren ^1,2,†^, Qiang Wang ^1,2,†^, Jianghua Chen ^1^, Hengwei Zhang ^1,2^, Zhoule Guo ^1^, Meijuan Xu ^1,2^, Xian Zhang ^1,2^*, Zhiming Rao ^1,2^

^1^ Key Laboratory of Industrial Biotechnology of the Ministry of Education, School of Biotechnology, Jiangnan University, Wuxi, Jiangsu 214122, China.

^2^ Yixing Institute of Food and Biotechnology Co., Ltd, Yixing, 214200, China.

* Correspondence to: Xian Zhang, zx@jiangnan.edu.cn

^†^ These authors contributed equally to this work.

**Table S1.** Strains and recombinant plasmids used in this work.

| plasmids and media | strains | source |
| --- | --- | --- |
| *Bacillus subtilis* |  |  |
| *B. subtilis* 168 | *trpC2* | Laboratory conservation |
| RK-E | *B. subtilis* 168Δ*sigE* | This experiment constructs |
| RKC-1 | *B. subtilis* 168Δ*lytC* | This experiment constructs |
| RKCE1 | *B. subtilis* 168Δ*lytC*Δ*sigE* | This experiment constructs |
| RKC-2 | *B. subtilis* 168Δ*sigD* | This experiment constructs |
| RKC-3 | *B. subtilis* 168Δ*pcfA* | This experiment constructs |
| RKC-4 | *B. subtilis* 168Δ*flgD* | This experiment constructs |
| RKC-5 | *B. subtilis* 168Δ*xpf* | This experiment constructs |
| RKC-6 | *B. subtilis* 168Δ*spo0A* | This experiment constructs |
| RKC-7 | *B. subtilis* 168Δ*skfA* | This experiment constructs |
| RKC-8 | *B. subtilis* 168Δ*sdpC* | This experiment constructs |
| RKC-9 | *B. subtilis* 168Δ*spollE* | This experiment constructs |
| RKC-10D | *B. subtilis* 168Δ*lytC*Δ*sigD* | This experiment constructs |
| RKC-11T | *B. subtilis* 168Δ*lytC*Δ*sigD*Δ*pcfA* | This experiment constructs |
| RKCE11 | *B. subtilis* 168Δ*sigE*Δ*lytC*Δ*sigD*Δ*pcfA* | This experiment constructs |
| RKC-12Q | *B. subtilis* 168Δ*lytc*Δ*sigD*Δ*flgD*Δ*pcfA* | This experiment constructs |
| RKR-13 | *B. subtilis* 168Δ*yluC* | This experiment constructs |
| RKR-14 | *B. subtilis* 168Δ*mraZ* | This experiment constructs |
| RKRE14 | *B. subtilis* 168Δ*mraZ*Δ*sigE* | This experiment constructs |
| RKCR-15T | *B. subtilis* 168Δ*lytC*Δ*sigD*Δ*mraZ* | This experiment constructs |
| RKCRE15 | *B. subtilis* 168Δ*sigE*Δ*lytC*Δ*sigD*Δ*mraZ* | This experiment constructs |
| CE1TG | *B. subtilis* 168Δ*sigE*Δ*lytC* /pMA5-*glsA* | This experiment constructs |
| CE11TG | *B. subtilis* 168Δ*sigE*Δ*lytC*Δ*sigD*Δ*pcfA*/pMA5-*glsA* | This experiment constructs |
| RE14TG | *B. subtilis* 168Δ*sigE*Δ*mraZ*/ pMA5-*glsA* | This experiment constructs |
| CRE15TG | *B. subtilis* 168Δ*sigE*Δ*lytC*Δ*sigD*Δ*mraZ*/ pMA5-*glsA* | This experiment constructs |
| CE11A | *B. subtilis* 168Δ*sigE*Δ*lytC*Δ*sigD*/pMA5- R137F | This experiment constructs |
| CRE15A | *B. subtilis* 168Δ*sigE*Δ*lytC*Δ*sigD*Δ*mraZ*/ pMA5- R137F | This experiment constructs |
| pMA5-glsA | Kan^r^，Overexpression of the *glsA* gene | This experiment constructs |
| pMA5-R137F | Expression of Spase mutant *gtfA* R137F in *B. subtilis*, Kan^r^ | Laboratory conservation |
| PDG148 | Kan^r^ ，Temperature-sensitive plasmids containing crease | Laboratory conservation |

**Table S2.** Proportion of viable cells in stable growth phase in harmful substances.

| Types/ /concentrations | 0g·L^-1^ | 0.5g·L^-1^ | 1.0g·L^-1^ | 1.5g·L^-1^ | 2.0g·L^-1^ | 2.5g·L^-1^ | 3.0g·L^-1^ |
| --- | --- | --- | --- | --- | --- | --- | --- |
| potassium nitrate | 100 % | 91.6 % | 83.4 % | 79.5 % | 72.2 % | 70.0 % | 62.1 % |
| furfural | 100 % | 89.4 % | 87.1 % | 80.3 % | 70.9 % | 61.8 % | 54.9 % |

Determine the number of viable cells by spreading on LB plates.

**Fig. S1** Production of L-glutaminase and OD_600_ of strains in the 5 L fermenter during 96 h fed-batch biotransformation.


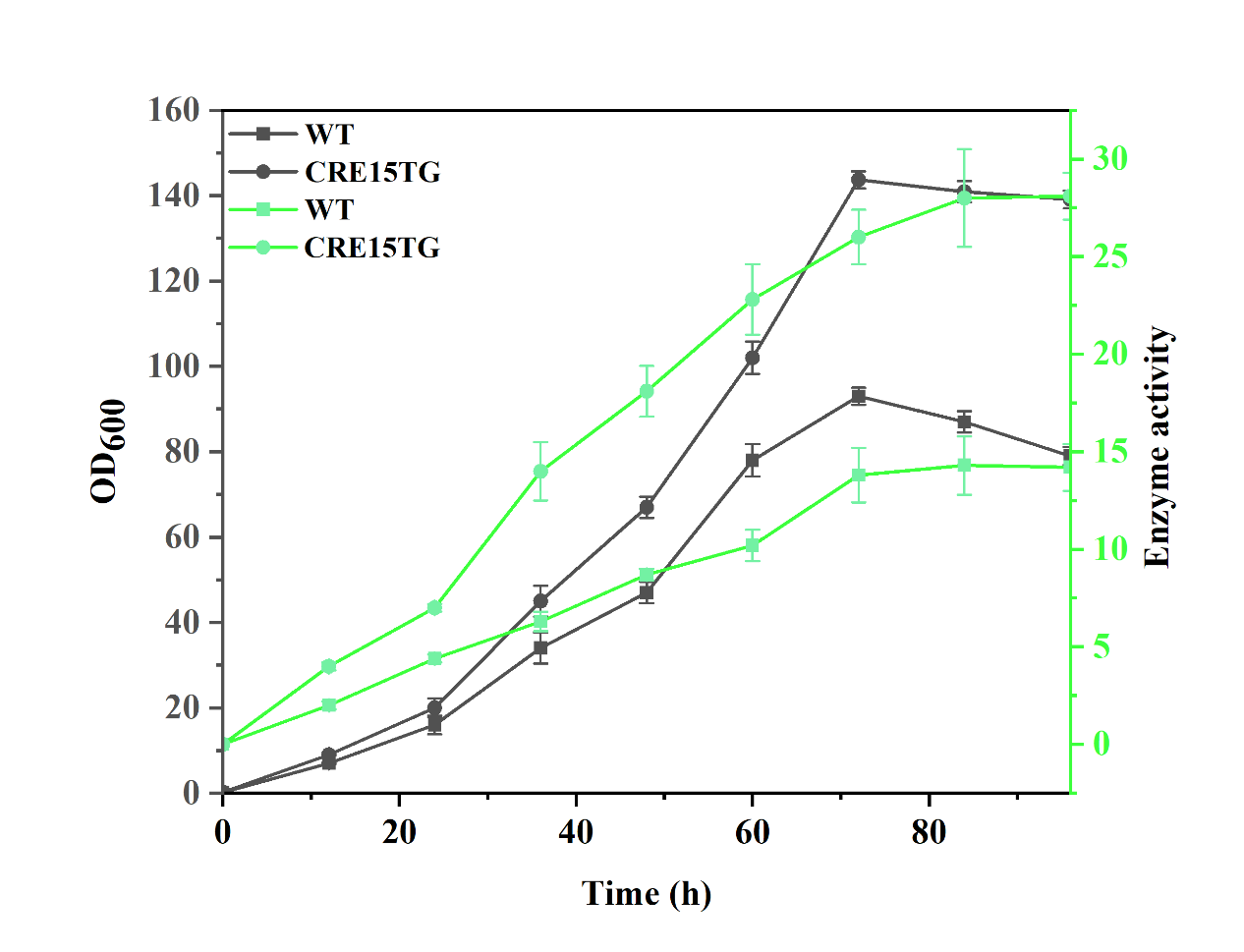


**HIGHLIGHTS**

1. Lifespan engineering strategy can be used to construct highly robust and productive microbial chassis cells.
2. The manipulation of cell lifespan can alter cell morphology and physiological properties such as tolerance to toxic substances.

3. Systematic modification of cell lifespan is an option for future engineering of industrialization *B. subtilis.*
